# Case Report: Echinocandin-Resistance *Candida glabrata FKS* Mutants From Patient Following Radical Cystoprostatectomy Due to Muscle-Invasive Bladder Cancer

**DOI:** 10.3389/fonc.2021.794235

**Published:** 2021-12-15

**Authors:** Maria Szymankiewicz, Krzysztof Kamecki, Sylwia Jarzynka, Anna Koryszewska-Bagińska, Gabriela Olędzka, Tomasz Nowikiewicz

**Affiliations:** ^1^ Department of Microbiology, Prof. F. Łukaszczyk Oncology Centre, Bydgoszcz, Poland; ^2^ Department of Urological Oncology, Prof. F. Łukaszczyk Oncology Centre, Bydgoszcz, Poland; ^3^ Department of Medical Biology, Medical University of Warsaw, Warsaw, Poland; ^4^ Department of Surgical Oncology, Nicolaus Copernicus University Ludwik Rydygier’s Collegium Medicum, Bydgoszcz, Poland; ^5^ Department of Clinical Breast Cancer and Reconstructive Surgery, Prof. F. Łukaszczyk Oncology Centre, Bydgoszcz, Poland

**Keywords:** *Candida glabarta*, echinocandins, antifungal resistance, *FKS* mutation, bladder cancer cystoprostatectomy

## Abstract

Invasive *Candida glabrata* infections are not common complications after radical cystoprostatectomy. Furthermore, resistance to echinocandins arising during the course of a patient’s treatment is rarely recognised. We described a case of development of echinocandin resistance in a patient with muscle-invasive bladder cancer (pT2b N0 M0, high grade) diagnosis, subjected to radical cystoprostatectomy and exposed to echinocandins. A male patient with a previous surgical history after a traffic accident, who was operated on due to bladder cancer, underwent an episode of candidemia and mixed postoperative wound and urinary tract infection caused by *C. glabrata* and extended spectrum β-lactamase (ESBL)-producing *Escherichia coli* during hospital treatment. The patient was started on caspofungin. Repeat blood cultures showed clearance of the bloodstream infection; however, infection persisted at the surgical site. Resistance to echinocandins developed within 2 months from the day of initiation of therapy with caspofungin in the *C. glabrata* strain obtained from the surgical site. The isolates sequentially obtained during the patient’s treatment demonstrated resistance to echinocandins due to the mutation in hotspot 1 *FKS2*. Although resistance to echinocandins is relatively rare, it should be considered in oncological patients with increased complexity of treatment and intestinal surgery.

## Introduction

The changing distribution of *Candida* species responsible for infections, with the predomination of *Candida glabrata*, significantly limits therapeutic options in oncological patients (1, 2). The first-line treatment of invasive infections includes echinocandins, such as *C. glabrata*, which present a low susceptibility to azoles (3). However, resistance to echinocandins has also been rising over the past decade. In some studied populations, especially in the USA, the rate of resistant isolates among this species exceeded 12.0% (4). In Europe, the prevalence of echinocandin-resistant *C. glabrata* is lower and reported to be 1.5% (5). One-third of echinocandin-resistant isolates may be multidrug resistant (6). As a consequence, infections due to *C. glabrata* pose a threat to patients, are difficult to treat, and are associated with a high mortality. Resistance to echinocandins due to a mutation in one or two “hot spot” regions of the *FKS1* or *FKS2* genes, encoding a subunit of the 1,3-β-D glucan synthase protein (the potential target enzyme echinocandins), is a serious clinical problem, resulting in echinocandin treatment failure and poor clinical outcomes (7). *FKS* genes are necessary for production of 1,3-β-D-glucan, a component of the *Candida* cell wall and the target of echinocandins, such as micafungin, anidulafungin, and caspofungin. Unlike amphotericin or azoles, these drugs are the safest for patients and most active on a broad spectrum for pathogenic fungal strains. The acquisition of resistance is often favoured by intra-abdominal candidiasis, repeat operations of the gastrointestinal tract, and poor drug penetration (3, 8). Infection can also be complicated by multidrug-resistant bacterial coinfection, which makes treatment even more difficult (9). We describe a case of rapid development of echinocandin resistance in a patient with an episode of candidemia and mixed postoperative wound and urinary tract infection caused by *C. glabrata* and extended spectrum β-lactamase (ESBL)-producing *Escherichia coli*.

## Description of Patient

**Figure 1:**
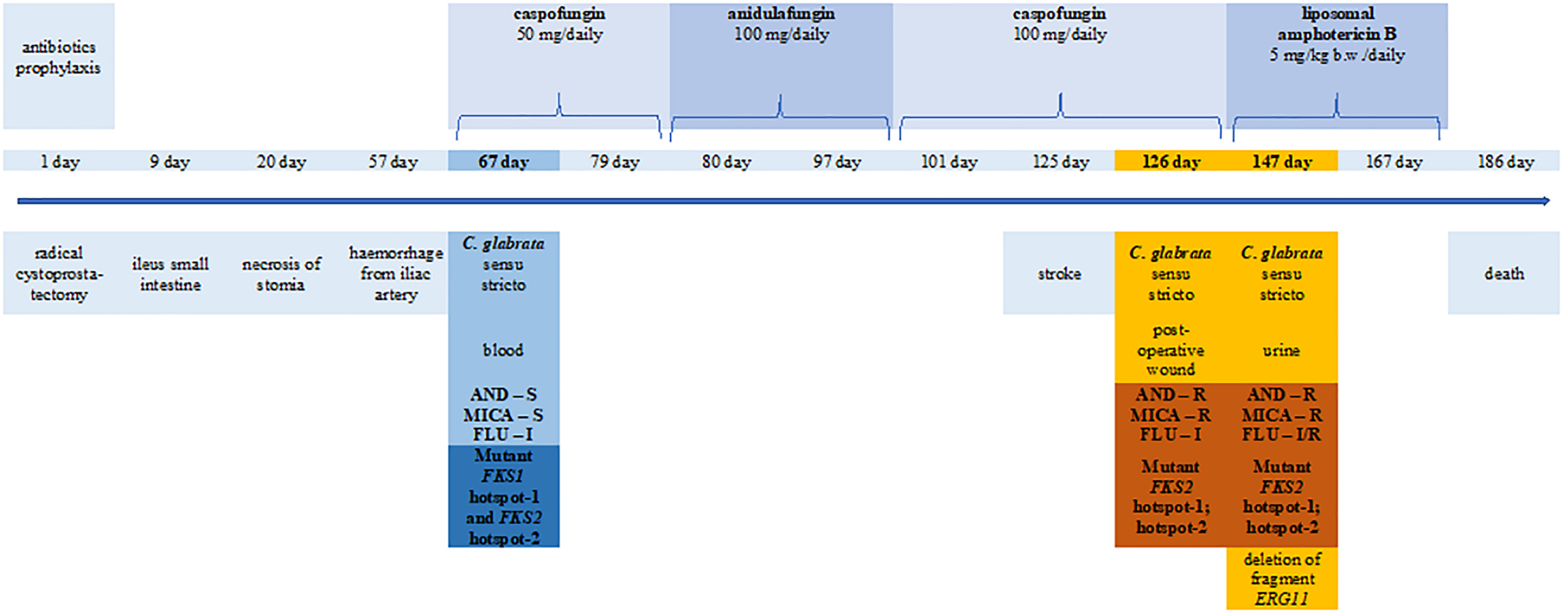
Timeline of the clinical procedures performed in patients with muscle-invasive bladder cancer during *Candida glabrata* infection. AND, anidulafungin; MICA, micafungin; FLU, fluconazole.

This work describes the case of a 66-year-old male patient who underwent surgery due to a muscle-invasive bladder cancer (pT2b N0 M0, high grade) and was subjected to radical cystoprostatectomy with a permanent, incontinent diversion of urine to the abdominal skin using a separated piece of small intestine as a stoma (ileal conduit). The course of the operation was difficult due to multiorgan traffic injury of the abdomen demanding urgent surgical treatment a few years prior. During the radical cystoprostatectomy, there was a need to dissolve many adhesions, remove ischaemic tissues, and partially resect the small bowel. Surgery was performed under antibiotic prophylaxis: ceftriaxone, 1 g every 12 h for 3 days and metronidazole, 0.5 g every 8 h for 3 days. On the eighth day of treatment, eventration and ileus occurred, requiring urgent surgery (to dissolve adhesions of the small intestine). An ileus recurred on day 11, and during the surgery, necrosis of the stoma was evident. The ischaemic tissues were removed, the ureters recatheterised, and bilateral percutaneous nephrostomies were performed. Wound healing was complicated by skin necrosis, which required several surgical treatments. A small intestine content appeared in the wound on day 20. Severe haemorrhage from the iliac artery occurred on day 57 and after urgent surgery, lower limb ischaemia appeared. The failure of treatment required a limb amputation at the thigh level. Ischaemic changes of the postoperative wounds and lower limb amputation stump progressed in the further course of hospitalisation. Dialysis was performed due to renal failure. On day 125, a stroke occurred. The patient died on day 186 after the primary surgery of excision of the urinary bladder as a result of gradually developing multiorgan failure (**Figure 1**).

## Diagnostic Assessment and Results

### Microbiological Monitoring

The patient was monitored microbiologically throughout the hospital stay. Blood, material from the postoperative wound, abscesses, urine, and peritoneal fluid were cultured. Three fungal isolates collected during the course of treatment were analysed. All examined strains were isolated during routine diagnostic procedures and then characterised by phenotypic and genotypic methods (**Supplementary Material Data**). The clinical cultures were performed on Sabouraud glucose agar. Species identification was performed using VITEK 2 YST ID card and multiplex polymerase chain reaction using a blood culture identification in the FilmArray system. The microorganism was identified as *C. glabrata.* All clinical isolates were reidentified by MALDI-TOF mass spectrometry.

On day 67, after the primary surgery, positive blood culture for *C. glabrata* was reported. All blood cultures drawn after 10 days from a positive result remained negative for *Candida* until the patient’s death. Ten days after the positive blood result, during caspofungin therapy, other fungal infections occurred. We detected *C. glabrata* in the postoperative wound (on day 126) and urine (nephrostomy, on day 147) with different susceptibility patterns as the isolate originated from the blood. Echinocandin-resistant isolate was detected in a wound sample at day 49 from the first positive result from this infection site, and on day 59 after initiation of therapy with caspofungin. At that time, we also isolated a phenotypically similar strain from urine. Both the postoperative wound and urine isolates were recovered from the same surgical site. The strain isolated from urine at day 80, after starting antifungal treatment with caspofungin, was subjected to molecular testing.

Additionally, the microorganisms inhabiting the digestive tract, specifically *E. coli*, *Enterococcus faecium*, *Enterococcus faecalis*, *Bacteroides thetaiotaomicron*, and *Pseudomonas aeruginosa* were isolated. *E. coli* recovered from the postoperative wound infections was identified as ESBL-*E. coli*, which is multidrug resistant and described in our previous work as belonging to the B2 phylogenetic group and carrying *bla*_CTX-M-15_ gene with a unique pulsed field gel electrophoresis pattern (10). As a result, a number of antibiotics, including those that are broad-spectrum, were ordered.

### Antifungal Susceptibility

Antifungal susceptibility was determined by using Micronaut-AM EUCAST AFST 2-Test Plate, which was based on the broth microdilution procedure, as recommended by the manufacturer. Drug susceptibility was assessed according to The EUCAST antifungal clinical breakpoint table (11, 12).

The isolate from the blood sample was susceptible to all examined antifungal echinocandins (micafungin, anidulafungin) and amphotericin B; susceptibility to fluconazole was intermediate/susceptible with increased exposure. Except for resistance to echinocandins, the isolates recovered from the postoperative wound and urine samples were intermediate/increased exposure susceptible to fluconazole (minimal inhibitory concentration (MIC), 8 mg/L) and susceptible to amphotericin B (MIC, 0.5 mg/L) (**Table 1**). According to the epidemiological cutoff values (EVCs), isolates susceptible to anidulafungin with micafungin (MIC of 0.03 mg/L) did not harbour a *FKS* mutation conferring resistance to echinocandins. In addition, the susceptibility to fluconazole in the strain obtained from urine was assessed using gradient strips impregnated with fluconazole with *E*-test system. The interpretation criteria used is outlined above. Using this method, the *C. glabrata* strain showed fluconazole resistance (MIC, 64 mg/L).

**Table 1:** Antifungal susceptibility for *Candida glabrata* isolates originated from the same patient.

Isolate	Origin of the isolate	MIC value of antifungal agents (mg/L)			
		Amphotericin B	Fluconazole	Micafungin	Anidulafungin
1	Blood sample	0.5	8.0	0.0078	0.015
2	Postoperative wound sample	0.5	8.0	**0.0625**	**0.125**
3	Urine sample (nephrostomy)	0.5	8.0	**0.25**	**0.5**
1/2/3	Interpretation with EUCAST antifungal clinical breakpoints table v. 9.0	S/S/S	IM/IM/IM	S/R/R	S/R/R
1/2/3	Interpretation with EUCAST antifungal clinical breakpoints table v. 10.0	S/S/S	I/I/I	S/R/R	S/R/R

MIC, minimum inhibitory concentration; S, susceptible; R, resistant; IM, intermediate. I, susceptible, increased exposure. Isolates susceptible to anidulafungin with micafungin MIC of 0.03 mg/L do not harbour a FKS mutation conferring resistance to the echinocandins, according to the new EUCAST antifungal recommendation (v.10.0). Echinocandin resistance - black bold.

### DNA Manipulations and *FKS* Sequencing

Identification of the *Candida* isolates was confirmed by PCR using specific ITS1 and ITS4 primers directed against the panfungal internal transcribed spacer ITS1-5.8S-ITS4 region and further were digested with restriction enzymes (13, 14). A multiplex mPCR-ID assay identified the clinical isolates as *C. glabrata* sensu stricto and analysed by random amplified polymorphic DNA (**Supplementary Figure S1**) (15, 16).

The main goal of genotyping analysis was to detect *FKS1* and *FKS2* (β-D-1,3-glucan synthase complex) genes, and sequencing to evaluate the prevalence of hotspot-1 and hotspot-2 mutations. In this study, a search for mutations was performed by the PCR method (**Supplementary Materials Data**) (17–22). Nucleotide and deduced amino acid sequences obtained for beta-1,3-glucan synthase catalytic subunit Fks1 and Fks2 with hotspot-1 and hotspot-2 regions were compared with corresponding sequences of the *C. glabrata* ATCC90030 echinocandin-susceptible wild-type reference strain (GeneBank accession number HM366440 and HM3664442 for *FKS1* and *FKS2*, respectively).

Only one strain, the *C. glabrata* isolate from blood, in which the phenotypic studies turned out to be sensitive against echinocandins, displayed Fks1 substitutions. The substitutions were in positions L628, S629, L630, and D632, of which S629 and D632 mutations generated amino acid substitutions, from serine to phenylalanine and from aspartic acid to alanine, respectively (**Table 2**). The mutations detected in the *FKS1* hotspot-1 may be spontaneous and related to clinical echinocandin exposure (7, 23, 24). None of the analysed *C. glabrata* clinical isolates displayed *FKS1* hotspot-2 region mutations. In all the clinical strains isolated from the postoperative wound and urine samples that were echinocandin-resistant, mutations in *FKS2* hotspot-1 and *FKS2* hotspot-2 were identified (**Table 2**). A single nucleotide changes in the *FKS2* hotspot-1 of the wound isolate resulting in a leucin to tryptophan substitution at position 662 was identified. In the same region of the urine isolate, the deletion of three nucleotides leading to the removal of phenylalanine from Fks2 protein was observed. This mutation may result in the altered structure of the Fks2 protein and/or lack of its activity, which explains the negative response to the patient’s further treatment. In addition, one additional mutation in a *FKS2* hotspot-2 region, namely R1377, leading to substitution of arginine to lysine, was detected for all clinical strains. Mutations in *FKS2* could characterise echinocandin-resistant *C. glabrata* strains, especially in the hotspot-1 fragment. According to literature data, the mutations detected in the *FKS1* hotspot-1 positions S629, *FKS2* hotspot-1 positions F659del, and *FKS2* hotspot-2 position R1377 may play an important role in rise of echinocandin-resistant mutants (18–22, 25).

**Table 2:** Mutations in hotspot areas of FKS1 and FKS2.

Isolate	FKS1 hotspot-1 (625-633)	FKS1 hotspot-2 (1340-1347)	FKS2 hotspot-1 (659-667)	FKS2 hotspot-2 (1374-1381)
Candida glabrata	FLILSLRDP	DWVRRYTL	FLILSLRDP	DWIRRYTL
ATCC 90030				
Blood	FLILFLRAP	DWVRRYTL	FLILSLRDP	DWIKRYTL
	**L628**			**R1377(Arg→Lys)**
	**S629(Ser→Phe)**			
	**L630**			
	**D632(Asp Ala)**		FLIWSLRDP	DWIKRYTL
Postoperative wound	FLILSLRDP	DWVRRYTL	L662(Leu→Trp)	**R1377(Arg→Lys)**
Urine	FLILSLRDP	DWVRRYTL	*LILSLRDPI	DWIKRYTL
			**F659del**	**R1377(Arg→Lys)**

Echinocandin-sensitive isolate—blood; echinocandin-resistant isolates—postoperative wound, urine; substitution nucleotide with changes in amino acid—red bold; substitution nucleotide without changes in amino acid—green bold; amino acid: Ser, serine; Phe, phenylalanine; Asp, aspartic acid; Ala, alanine; Arg, arginine; Lys, lysine; Leu, leucin; Trp, tryptophan. Deletion of amino acid and frameshift—blue*; mutations —black bold, mutations associated with high echinocandin resistance when altered —black bold with gray shading.

Additionally, the epidemiology of fluconazole resistance and other azole resistance (indirectly) was performed with the detection of *ERG11* and *PDR1* genes (pleiotropic drug resistance). The product of the *ERG11* gene leads to inhibition of demetylase needed for ergosterol biosynthesis (22, 26). The *PDR1* gene acts as a regulator of the ATP-binding cassette (ABC), relevant in the drug efflux process and mitochondrial dysfunction by *Candida* species in the susceptibility to azoles (27, 28). The PCR reaction for *EGR11* gene was present in the genomes of all tested clinical strains; however, with the second pair of ERG2 primers (**Supplementary Table S1**), no product was achieved from the urine samples (**Supplementary Figure S1**). This, in turn, may indicate a fragment deletion in the *ERG11* gene from the *C. glabrata* genome isolated from the urine sample. This observation requires further studies, but undoubtedly, previous exposure to fluconazole could cause mutations in the *ERG11* gene, especially the deletion observed in other studies (14, 22, 27). An additional *E*-test assay performed as an alternative method for the urine isolate resulted in a MIC value of 64 mg/ml. This value may indicate resistance to fluconazole, and thus confirm the deletion in the *ERG11* gene in this isolate. In addition, other authors have reported inconsistencies in the results of broth microdilution and the *E*-test method for echinocandin-resistant *C. glabrata* strains that were not observed for susceptible strains (22, 29). The *PDR1* gene was identified in all clinical isolates by PCR assay, which may result in a reduced susceptibility to fluconazole (14, 27).

## Antifungal Treatment

The patient was prophylactically treated with fluconazole from March 10 to 25, 2019 and from April 22 to 27, 2019, receiving a single intravenous (IV) dose of 200 mg once daily. On April 28, 2019 (day 67), the patient was switched from fluconazole to caspofungin, which was maintained until May 9 at a dose of 50 mg daily (70 mg loading dose on the first day). Anidulafungin was then administered from May 10 to 28, 2019 (79th–97th day of stay), a single IV loading dose of anidulafungin 200 mg on day 1, followed by 100 mg once daily and caspofungin from June 01 to 26, 2019 (101st–126th day of stay), as described above. On July 17, 2019 (day 147), the patient was started on liposomal amphotericin B (L-AmB) at a dose of 5 mg/kg of body weight daily and continued until August 06, 2019 (day 167).

## Discussion

Growing resistance to antifungal drugs is becoming a serious challenge, especially among oncological patients. The phenomenon of resistance build-up during the treatment of *Candida* infections is of particular concern. We reported a case of a patient with muscle-invasive bladder cancer and candidemia episode, caused by *C. glabrata* susceptible to echinocandins and later *C. glabrata* infection of the postoperative wound and urinary tract complicated by infection with multidrug-resistant *E. coli.* We have demonstrated that isolates sequentially obtained from the analysed patient during the patient’s treatment developed resistance to echinocandins due to a *FKS2* hotspot-1 mutation.

The combination of multiple risk factors in our patient with a solid tumour of the bladder presented a high risk for *Candida* infections. The main risk factors were the complexity of surgery, reoperations, exposure to broad-spectrum antibiotics in the course of multidrug-resistant bacteria infection treatment (ESBL-*E. coli*), multiple blood transfusions, and older age. Moreover, a stay in the intensive care unit and a prolonged hospital stay also could favour *Candida* infection, as previously reported (30, 31). Most significant, however, was the patient’s previous surgical treatment history, which resulted in a large extent of ischaemic intestinal lesions after radical cystoprostatectomy.

In accordance with the adopted strategy of managing infections in our hospital, the patient was monitored microbiologically and the antifungal therapy was selected according to the etiological agents of infection and susceptibility patterns. Moreover, in the case of candidemia, the use of a genetic method-multiplex PCR for the detection of microorganisms in the blood allowed for the implementation of antifungal treatments appropriate to species identification before the assessment of drug susceptibility. The proceedings were consistent with the current literature data, as the delay in administration of antifungals in candidemia is associated with a higher risk of therapeutic failure among patients with candidemia (32, 33).

Candidemia treatment, first with caspofungin and then with anidulafungin, resulted in a positive response without any new positive episode of candidemia during the period of hospitalisation. However, during therapy, other fungal infections were recognised at the surgical site. For that reason, prolonged and repeated antifungal treatment with caspofungin was initiated and continued for 26 days. First, an echinocandin-resistant isolate was detected in the wound sample within 2 months of initiation of caspofungin therapy. At that time, a phenotypically similar strain from the urine was also isolated. Both the postoperative wound and urine isolates were recovered from the same surgical site. Clinical trials show that >5 days of antifungal therapy (especially high-dose caspofungin, >100 mg) was directed to the occurrence of *FKS* mutations, especially in isolates from the urine tract (22, 34, 35).

It is extremely important to determine the EVCs for echinocandins that may be a phenotypic indicator for the occurrence of Fks mutations. Some authors suggest an EVC of 0.25 mg/L for caspofungin, 0.12 mg/ml for anidulafungin and 0.03 mg/ml for micafungin (17, 20, 35). Similarly, in our study, a MIC of 0.125 mg/ml for anidulafungin and 0.06 mg/ml for micafungin resulted in mutations in *FKS2*. Micafungin and anidulafungin could play important roles as markers of *FKS* mutations (21, 22, 35).

In our case report, we demonstrated that the isolates obtained from the surgical site showed increased MIC values of echinocandins that rendered them echinocandin resistant (anidulafungin and micafungin were examined in this study), consistent with the EUCAST antifungal clinical breakpoint table v. 9.0 and table v. 10. According to the last EUCAST recommendation (table v.10), isolates with micafungin MIC >0.03 mg/L are considered to be resistant due to harbouring a *FKS* mutation and Fks sequencing is recommended. In our case, molecular testing confirmed resistance to echinocandins by hotspot-1 *FKS2* mutation. The isolate originating from urine demonstrated amino acid deletion F659del, which is most common in *C. glabrata* and is associated with resistance and poor prognosis (21, 36, 37). This isolate showed the highest MIC value for echinocandins among the examined isolates. The isolate recovered from the postoperative wound possessed mutation L662 and was demonstrated at low-level resistance for echinocandins (MIC for anidulafungin, 0.125 mg/L; for micafungin, 0.06 mg/L). In the blood isolate, no effect of the S629 mutation in the hotspot-1 region of the *FKS1* gene regarding reduced sensitivity to echinocandins was observed in this analysed case. This result was confirmed in many studies that reported *Candida* strains susceptible to echinocandins with mutations S629 in the *FKS1* hotspot-1 (21, 29, 35). Approximately 80% of susceptible strains could present changes in the genotype (21). These results were reported by Aldejohann et al. (29), who tested these same echinocandin-susceptible strains with a few mutations independently in eight different labs in Germany. In contrast, in the study by Castanheira et al. (37), the isolates that displayed this mutation presented resistance to echinocandins.

Prior exposure to antifungal agents, i.e., prophylaxis with fluconazole before candidemia (for a total of 22 days), probably influenced the selection of *C. glabrata* with naturally reduced azole susceptibility. In addition, prior treatment with caspofungin (for a total of 37 days) influenced the selection pressure for *C. glabrata*, with increasing MICs for echinocandins and subsequent resistance to this group of antifungals. Echinocandin-susceptible isolates without mutation in hotspot-1 *FKS*2 gene in the study patient was detected before the isolation of resistant isolates and may indicate the acquisition of resistance during treatment, as reported in other studies (4). Mutations in the FKS1 hotspot-1 in amino acid positions 625 or 632 and in the *FKS2* hotspot-1 in positions 659, 662, or 663 were found in *C. glabrata* resistant to new antifungal drugs (for example, ibrexafungerp), which was investigated by Arendrup et al. (38), and has evolved into a clinical problem for next decades.

It seems that the most likely source of the candidemia episode in our patient was the gastrointestinal tract due to the possible imbalance of the intestinal microbiome, a large extent of intestinal necrosis, damaged mucosal tissue, and intestinal fistulas and intestinal leakage, as previously observed by other authors (34, 39). As a consequence, the local condition of the abdominal cavity likely reduced drug penetration. In turn, poor drug penetration and the persistence of prolonged subinhibitory echinocandin levels could influence selection pressure and the emergence of resistance (3, 9). Coexistence in surgical site infection of *C. glabrata* and multidrug-resistant bacteria (ESBL-producing *E. coli*) points to a significant problem among patients with solid tumours and emphasises the importance of the gastrointestinal tract as a reservoir of multidrug-resistant microorganisms in cancer patients. Finally, antifungal treatment with liposomal amphotericin B eventually led to microbiological success. Unfortunately, the ESBL-positive infection persisted, despite targeted treatment with carbapenems. Ultimately, further deterioration of the patient’s clinical condition led to death.

In conclusion, resistance to echinocandins in *C. glabrata* developed in a patient presenting with muscle-invasive bladder cancer and surgical history due to previous multiorgan traffic injury of the abdomen. Complexity of treatment and intestinal surgery should compel the treating physician to consider resistant *Candida* infection, although resistance to echinocandins in European countries is still rare.

Strength of the study:
1. The rare case report presenting *Candida glabrata* FKS mutants in patients with solid tumours.
2. In this case, apart from known mutations (S629; F659del), we described rare mutations in *FKS2* hotspot-1 (L662: leucin to tryptophan); in hotspot-2 (R1377: arginine to lysine); and in *FKS1* hotspot-1 (D632: aspartic acid to alanine), which led to changes in amino acids and protein properties, echinocandin resistance, and higher MICs values.
3. Correlation of echinocandin MIC values with the results of previous scientific research mapping Fks mutations within the hotspot conserved regions seems to be a good screening method in detecting clinical resistance of *C. glabrata* to echinocandins. This monitoring could be in a procedure algorithm in relation to all patients with intestinal surgery exposed to endogenous *C. glabrata* infections.
4. The presented results, especially estimation of the MICs and epidemiological cutoff values (EVCs) for all echinocandins that may be indicative of the presence of a mutation leading to clinical failure, could help in quickly detecting resistance and administering the appropriate doses of antifungal drugs.
Limitation of the study:
1. The substitutions of the nucleotides leading to changes in amino acid in the genes encoding glucan synthase (Fks) constitute only one mechanism that contributes to the lack of echinocandin interaction with the *C. glabrata* cell wall. There are many possible mechanisms for echinocandin resistance of *C. glabrata*, for example, exposure to echinocandins (also prophylaxis in risk groups); changes in cell wall composition (increase of chitin/mannan) related to the activation of *RHO1* gene and MAPK cascade dependent on calconeurine or regulation in the cell cycle; deletion of yapsin-like proteins in the cell wall; and changes in membrane sphingolipids. For this reason, whole genome or exome sequencing and searching for new resistance mechanisms should be the subject of future research on an appropriate statistically significant number of strains.

## Data Availability Statement

The original contributions presented in the study are included in the article/**Supplementary Material**. Further inquiries can be directed to the corresponding author.

## Ethics Statement

The studies involving human participants were reviewed and approved by the Bioethical Commission at the Collegium Medicum of Nicolaus Copernicus University in Toruń (KB approval number 453/2021). Written informed consent for participation was not required for this study in accordance with the national legislation and the institutional requirements. Written informed consent was obtained from the individual(s) for the publication of any potentially identifiable images or data included in this article.

## Author Contributions

MS was responsible for the conception and design of the study. MS, TN, and KK organised the database and resources. SJ and AK-B performed the genetic examinations and analysis. GO contributed to the interpretation of molecular results. MS, SJ, and AK-B wrote original draft preparation. MS and SJ wrote review and editing. MS provided supervision and project administration. All authors were involved in the analysis of findings, proved the manuscript, and contributed to writing. All authors read and approved the final version.

## Conflict of Interest

The authors declare that the research was conducted in the absence of any commercial or financial relationships that could be construed as a potential conflict of interest.

## Publisher’s Note

All claims expressed in this article are solely those of the authors and do not necessarily represent those of their affiliated organizations, or those of the publisher, the editors and the reviewers. Any product that may be evaluated in this article, or claim that may be made by its manufacturer, is not guaranteed or endorsed by the publisher.
