# Supplementary material for: Case Report: Echinocandin-Resistance Candida glabrata FKS Mutants From Patient Following Radical Cystoprostatectomy Due to Muscle-Invasive Bladder Cancer
Source: Front Oncol. 2021 Dec 15;11:794235. doi: 10.3389/fonc.2021.794235 (PMC8714647; doi:10.3389/fonc.2021.794235)
Supplement: Supplementary file 1 [file DataSheet_1.docx]

Supplementary Material

# Supplementary Data - Description of the phenotypic and molecular methods

## Phenotypic analysis

- Strains and culture conditions

The clinical cultures were performed on Sabouraud glucose agar (Oxoid, Basingstoke, UK) and Oxoid Brilliance Candida agar (Oxoid, Basingstoke, UK) at 35±1 0C in air for the first day, next at 25±1 0C in air for four days and 30±1 0C in air for three days. Species identification was performed using VITEK 2 YST ID card with VITEK 2 Compact (bioMérieux Inc., Hazelwood, Missouri, US). In a positive blood culture in an automated BacT/ALERT 3D system (bioMérieux Inc., Durham, NC, US), microbial genetic material has been additionally detected by multiplex polymerase chain reaction (PCR) using a blood culture identification panel (BCID) in the FilmArray system (BioFire Diagnostics, Salt Lake City, US), according to the manufacturer's instructions. The microorganism was identified as *C. glabrata*. Species identification was confirmed by positive cultures and identification as described above. All clinical isolates were reidentified by mass spectrometry. Proteomic identification by MALDI-TOF MS was performed using the IVD MALDI Biotyper Smart System, microflex LT/SH smart (Bruker Daltonik, Bremen, Germany).

- Antifungal Susceptibility Testing

Antifungal susceptibility was determined by using Micronaut-AM EUCAST AFST 2-Test Plate (Merlin Diagnostyka GmbH, Bornheim-Hersel, Germany), which was based on the broth microdilution procedure, as recommended by the manufacturer. *Candida parapsilosis* ATCC 22019 served as the control strain. Results were visually tested. Drug susceptibility was assessed according to The EUCAST antifungal clinical breakpoint table v. 9.0, valid from 2018-02-12 and compared with the newly recommended EUCAST antifungal clinical breakpoint table v. 10.0, valid from 2020-02-04 ([The European Committee on Antimicrobial Susceptibility Testing, 2018](#_ENREF_12); [The European Committee on Antimicrobial Susceptibility Testing, 2020](#_ENREF_13)). The susceptibility to fluconazole in the strain obtained from urine was assessed using gradient strips impregnated with fluconazole with E-test system (bioMérieux, Marcy l'Etoile, France) on Roswell Park Memorial Institute (RPMI) agar plates (bioMérieux, Marcy l'Etoile, France). The interpretation criteria used is outlined above.

## Molecular methods

- DNA manipulations and *FKS* sequencing

Genomic DNA from the isolates was extracted using the Syngen Fungi DNA Mini Kit (Syngen, Wroclaw, Poland) according to the manufacturer’s instructions. DNA quality and quantity was determined spectrophotometrically using the NanoDrop spectrophotometer DeNovix Ds-11 (DeNovix Inc., Wilmington, DE, US) and by agarose gel electrophoresis. RNA contamination was removed by RNaza A digestion (Blirt S.A., Gdansk, Poland). PCR reactions were performed in a Mastercycler Personal (Eppendorf, Hamburg, Germany) using specific primers (Supplementary Table 1). Reaction conditions were set individually depending on the primers and length of sequence to be amplified (Supplementary Materials). All enzymatic reactions were performed according to the manufacturer’s instructions. For the DNA fragment length analysis, the PCR products and DNA fragments generated by four restriction enzymes were subjected to electrophoresis according to standard procedures. The band patterns were visualised using the gel documentation ImageLab software (Bio-Rad, Inc., Hercules, CA, US) and photographed. Primer synthesis and DNA sequencing were performed by the DNA Sequencing and Oligonucleotide Synthesis Laboratory at IBB PAN (Warsaw, Poland). The *C. glabrata* ATCC2001 strain (CBS138) was run as a control in each experiment, while *Candida krusei* ATCCG258 and *C. parapsilosis* ATCC22019 were used in particular experiments.

- Identification of the *Candida* isolates

The panfungal internal transcribed spacer used was ITS1-5.8S-ITS4. The PCR reaction mix contained 2.5 µL of 10 pM of each primer, 10 µM deoxynucleotides, 5 µL 10X Ex Taq Buffer, 5 µL MgCl_2_ 20 mM, 0.25 µL TaKaRa Ex Taq proofreading polymerase (Takara Bio Inc., Shiga, Japan), and 2 µL DNA in a final volume of 50 µL. The PCR conditions were as follows: an initial denaturation phase at 94 °C for 5 min, followed by 32 cycles of denaturation phase at 94 °C for 30 s, annealing at 55 °C for 45 s, and extension at 72 °C for 1 min, with a final extension phase at 72 °C for 10 min ([Asadzadeh et al., 2019](#_ENREF_2); [Taei et al., 2019](#_ENREF_11)). The expected product size was of 871 bp obtained for all tested strains (data not shown).

- Characterisation of clinical isolates

PCR products amplified with ITS1 and ITS4 primers were digested with following restriction enzymes: EcoRI (Fermentas, Vilnius, Lithuania), HINDIII (Fermentas, Vilnius, Lithuania), BanII (Takara Bio Inc., Shiga, Japan) and MspI (Takara Bio Inc., Shiga, Japan). Digestions were carried out for each enzyme separately in a total volume of 50 μl. The reaction mixture, consisting of 25 μl of PCR-ITS product, 5 µl 10 x buffer, 5 µl BSA, and 2.5 µl enzyme, volume-adjusted with sterile distilled water, was incubated at 37 °C for 3 hours ([Mirhendi et al., 2011](#_ENREF_7)). Finally, 15 μl of each digest was electrophoresed. Fragments obtained after digestion of the PCR-ITS products of the clinical strains were consistent with those generated in the published data for *Candida glabrata* ATCC2001 strain (data not shown). Confirmation by a multiplex mPCR-ID assay using primers specific for *C. glabrata* sensu stricto (CGF), *C. nivarensis* (CNF) and *C. bracarensis* (CBF). The conditions for mPCR-PCR were 30 cycles, each cycle consisting of a denaturing step at 95 °C for 1 min, annealing at 52 °C for 30 s, extension at 72 °C for 1 min, with an initial denaturation step of 95 °C for 5 min and a final extension of 72 °C for 10 min. The final concentrations of 25 µL reactions were: 5 μL of 10 pmoles of each primer, 2.0 μL dNTP Mix 10 mM, 2.5 μL 10xEx Taq Buffer, 2.5 μL MgCl_2_ 20 mM, 0.25 μL TaKaRa Ex Taq proofreading polymerase (Takara Bio Inc., Japan), 2.0 μL of total genomic DNA and nuclease-free water ([Asadzadeh et al., 2019](#_ENREF_2); [Taei et al., 2019](#_ENREF_11)). As a quality control, reference strains of *C. glabrata* (ATCC 2001), *C. krusei* ATCCG258 and *C. parapsilosis* ATCC 22019 were used. The mPCR-amplified 360 bp product from all three analysed strains, as well as the *C. glabrata* ATCC2001 control strain, identified the clinical isolates as *C. glabrata* sensu stricto. A completely different amplicon and no amplicon were obtained from *C. krusei* ATCCG258 and *C. parapsilosis* ATCC22019 reference strains, respectively (Fig. 1). *Candida* isolates were analysed by random amplified polymorphic DNA using 1247 and 1290 short primers described by Olchawa et al. in 2013 ([Ergon and Gulay, 2005](#_ENREF_4); [Olchawa et al., 2013](#_ENREF_8)). The assays were performed in a total volume of 50µl, containing 2.0 μL of the genomic DNA from each strain (clinical isolate or control *C. glabrata* ATCC2001 strain), 2.5 μL of 10 pM of each primer, 2.0 μL dNTP Mix 10 mM, 2.5 μL RedTaq HotStar DNA 10Xbuffer, and 2.5 μL MgCl2 20 mM 0.25, μL polymerase RedTaq HotStar DNA (Merck KGaA, Darmstadt, Germany). After the initial denaturation step of 94 °C for 6 min, the amplification consisted of 40 cycles: 1 min at 94 °C, 1 min at 38 °C and 1 min at 72 °C, followed by a final extension of 6 min at 72 °C. An amount of 15 μl of each PCR product were analysed by gel electrophoresis (Supplementary Figure 1).

- Genotyping analysis to detect *FKS* mutations

In this study, the search for mutations was performed in the position of nucleotides (nt) specific for *FKS1* hotspot-1 in 625-633 nt, *FKS1* hotspot-2 in 1340-1347 nt, *FKS2* hotspot-1 in 659-667 nt and for *FKS2* hotspot-2 in 1374-1381 ([Zimbeck et al., 2010](#_ENREF_16); [Dudiuk et al., 2014](#_ENREF_3); [Khan et al., 2018](#_ENREF_5); [Kritikos et al., 2018](#_ENREF_6); [Rivero-Menendez et al., 2019](#_ENREF_9); [Al-Baqsami et al., 2020](#_ENREF_1)). The oligonucleotides primers (AF/AR, BF/BR, CF/CR, DF/DR) used for *FKS* hotspot evaluation are presented in Table 2. PCR for the identification and isolation of the mutation regions were performed in total volume of 100 μL using: 5 μL of 10 pM of each primer, 7.5 μL dNTP Mix 10 mM, 10 μL 10 x bufor, 10 μL MgCl_2_ 20 mM, 0.5 μL polymerase TaKaRa Ex Taq proofreading polymerase (Takara Bio Inc., Japan) and 3.0 μL of DNA. The amplification provided was as follows: 95 °C for 10 min, 35 cycle of denaturing at 95 °C for 1 min, annealing for primers AF/AR at 52 °C, BF/BR 51 °C, CF/CR 46 °C, DF/DR 49 °C for 30 s, extension at 72 °C for 30 s and final extension at 72 °C for 7 min. As a result, the amplicons for all clinical isolates and the reference *C. glabrata* ATCC 2001 strain were obtained ([Zimbeck et al., 2010](#_ENREF_16); [Dudiuk et al., 2014](#_ENREF_3); [Khan et al., 2018](#_ENREF_5); [Kritikos et al., 2018](#_ENREF_6); [Rivero-Menendez et al., 2019](#_ENREF_9); [Al-Baqsami et al., 2020](#_ENREF_1)). The oligonucleotide primers (AF/AR, BF/BR, CF/CR, DF/DR) used for FKS hotspot evaluation are presented in Supplementary Table 1.

- Sequencing

To detect mutations in the most active regions of FKS genes, obtained amplicons were purified with the DNA clean-up kit ExtractME (Blirt S.A., Gdansk, Poland) according to the manufacturer’s instructions. Finally, 12 purified PCR amplicons for FKS1 hotspot-1/hotspot-2 and FKS2 hotspot-1/hotspot-2 (four for each of the tested samples) were sequenced on both strands using the BigDye Terminator Mix v.3.1 chemistry (Applied Biosystems, Foster City, CA, USA), the ABI3730xl Genetic Analyser (Life Technologies, Carlsbad, CA, USA) and the same primers used for PCR amplification ([Zimbeck et al., 2010](#_ENREF_16); [Dudiuk et al., 2014](#_ENREF_3); [Khan et al., 2018](#_ENREF_5); [Kritikos et al., 2018](#_ENREF_6); [Rivero-Menendez et al., 2019](#_ENREF_9); [Al-Baqsami et al., 2020](#_ENREF_1)). Nucleotide and deduced amino acid sequences obtained for beta-1,3-glucan synthase catalytic subunit Fks1 and Fks2 with hotspot-1 and hotspot-2 regions were analysed using SnapGene 5.2.4. system (GSL Biotech LLC, San Diego, CA, USA).

- Epidemiology of fluconazole resistance

The PCR reaction for *EGR11* gene was based on two pairs of primers, ERG1-CG11F/CG11R and ERG2-CG11S3F/CG11S3R. The mixture in a total of 20 μL was performed using: 1 μL of 10 pM of each primer, 1.5 μL dNTP Mix 10 mM, 2 μL RedTaq HotStar DNA 10Xbuffer, 2 μL MgCl_2_ 20 mM, 0.1 μL polymerase RedTaq HotStar DNA (Merck KGaA, Darmstadt, Germany) and 2.0 μL of DNA. The amplification provided was as follows: 95 °C for 10 min, 33 cycle of denaturing at 95 °C for 1 min, annealing at 61 °C for 1 min, extension at 72 °C for 1.5 min and final extension at 72 °C for 7 min ([Xu et al., 2008](#_ENREF_15); [Al-Baqsami et al., 2020](#_ENREF_1)). The PCR reaction for *EGR11* gene was based on two pairs of primers, ERG1- CG11F/CG11R and ERG2-CG11S3F/CG11S3R. The *PDR1* gene was identified by PCR assay using a pair of CGPDR2S and CGPDR4AS primers, which resulted in the amplification of ~700 bp product. The reaction conditions used for amplification of *PDR1* gene were as follows: PCR mixture (20 μl) contained 1 μL of 10 pmoles of each primer, 1.5 μL dNTP Mix 10 mM, 2 μL 10 x bufor, 2 μL MgCl2 20 mM, 0.1 μL polymerase RedTaq HotStar DNA (Merck KGaA, Germany) and 2.0 μL of DNA. The amplification was performed using the following protocol: 95 °C for 10 min, 35 cycle of denaturing at 95 °C for 30 s, annealing at 53 °C for 1 min, extension at 72 °C for 1.5 min and final extension at 72 °C for 10 min ([Tsai et al., 2006](#_ENREF_14); [Simonicova and Moye-Rowley, 2020](#_ENREF_10)).

# Supplementary Figures and Tables


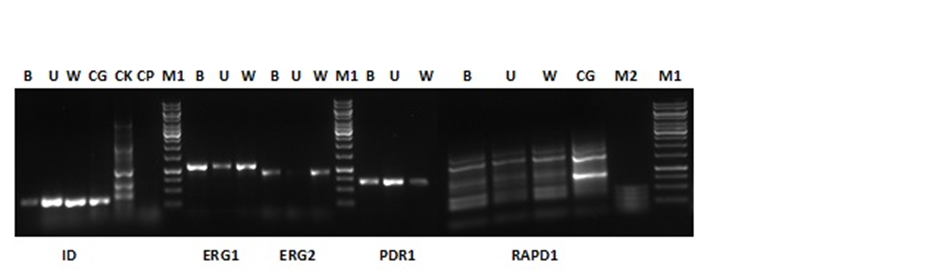


**Supplementary Figure 1.** Analysis of all PCRs products by agarose gel electrophoresis

Notes: *C. glabrata* strains from B – blood, U – urine, W – wound samples. M1 - DNA length standard marker - 1 kb ladders (Fermentas, Lithuania); M2 - DNA length standard marker - 500 bp ladders (Fermentas, Lithuania). ID – mPCR identification; ERG1 – PCR with CG11F/CG11R primers; ERG2 – PCR with CG11S3F/CG11S3R primers; PDR1 – PCR with CGPDR2S/CGPDR4AS primers; RAPD1 – PCR with 1247/1290 primers

**Supplementary Table 1.** Primers used in this study

| Primer | Sequence (5′ to 3′) | Product size (bp) | Trial |
| --- | --- | --- | --- |
| ITS1 | TCCGTAGGTGAACCTGCGG | ̴871 bp | PCR-ITS |
| ITS4 | TCCTCCGCTTATTGATATGC |  |  |
| CCR | CACGGAATTCTGCAATTCACA |  | mPCR-ID |
| CNF | GAGGAGTTTGTATCTTTCAACTT | ̴250 bp |  |
| CGF | CGGTTGGTGGGTGTTCTGC | ̴360 bp |  |
| CBF | TATTTACAAACTTTGTCAGAACTTA | ̴180 bp |  |
| 1247 | AAGGACCCGT |  | PCR-RAPD1 |
| 1290 | GTGGATGCGA |  |  |
| CG11F | TCCACCTCGAACCCGTATA | ̴1179-1667 bp | PCR-ERG1 |
| CG11R | TCCATGTTGATATCCACGATGACT |  |  |
| CG11S3F | GACGTGAGAAGAACGATATCCA | ̴1000 bp | PCR-ERG2 |
| CG11S3R | ATCAAGACACCAATCAATAGGTT |  |  |
| CGPDR2S | TATCCTAAGTATGGACAACG | ̴700 bp | PCR-PCDR1 |
| CGPDR4AS | GATTCCTTAAGCCCGATAAG |  |  |
| AF | CCATTGGGTGGTCTGTTCACG | ̴600-750 bp | PCR-*FKS1* hotspot-1 |
| AR | GATTGGGCAAAGAAAGAAATACGAC |  |  |
| BF | GGTATTTCAAAGGCTCAAAAGGG | ̴750-1000 bp | PCR-*FKS1* hotspot-2 |
| BR | ATGGAGAGAACAGCAGGGCG |  |  |
| DF | GCTTCTCAGACTTTCACCG | ̴600-700 bp | PCR-*FKS2* hotspot-1 |
| DR | CAGAATAGTGTGGAGTCAAGACG |  |  |
| CF | TCTTGACTTTCTACTATGCG | ̴750-1000 bp | PCR-*FKS2* hotspot-2 |
| CR | CTTGCCAATGTGCCACTG |  |  |

Abbreviations: F – forward primer, R – reverse primer

# References

Al-Baqsami, Z.F., Ahmad, S., and Khan, Z. (2020). Antifungal drug susceptibility, molecular basis of resistance to echinocandins and molecular epidemiology of fluconazole resistance among clinical Candida glabrata isolates in Kuwait. *Sci Rep* 10(1)**,** 6238. doi: 10.1038/s41598-020-63240-z.

Asadzadeh, M., Alanazi, A.F., Ahmad, S., Al-Sweih, N., and Khan, Z. (2019). Lack of detection of Candida nivariensis and Candida bracarensis among 440 clinical Candida glabrata sensu lato isolates in Kuwait. *PLoS One* 14(10)**,** e0223920. doi: 10.1371/journal.pone.0223920.

Dudiuk, C., Gamarra, S., Leonardeli, F., Jimenez-Ortigosa, C., Vitale, R.G., Afeltra, J., et al. (2014). Set of classical PCRs for detection of mutations in Candida glabrata FKS genes linked with echinocandin resistance. *J Clin Microbiol* 52(7)**,** 2609-2614. doi: 10.1128/JCM.01038-14.

Ergon, M.C., and Gulay, Z. (2005). Molecular epidemiology of Candida species isolated from urine at an intensive care unit. *Mycoses* 48(2)**,** 126-131. doi: 10.1111/j.1439-0507.2004.01086.x.

Khan, Z., Ahmad, S., Mokaddas, E., Meis, J.F., Joseph, L., Abdullah, A., et al. (2018). Development of Echinocandin Resistance in Candida tropicalis following Short-Term Exposure to Caspofungin for Empiric Therapy. *Antimicrob Agents Chemother* 62(4). doi: 10.1128/AAC.01926-17.

Kritikos, A., Neofytos, D., Khanna, N., Schreiber, P.W., Boggian, K., Bille, J., et al. (2018). Accuracy of Sensititre YeastOne echinocandins epidemiological cut-off values for identification of FKS mutant Candida albicans and Candida glabrata: a ten year national survey of the Fungal Infection Network of Switzerland (FUNGINOS). *Clin Microbiol Infect* 24(11)**,** 1214 e1211-1214 e1214. doi: 10.1016/j.cmi.2018.05.012.

Mirhendi, H., Bruun, B., Schonheyder, H.C., Christensen, J.J., Fuursted, K., Gahrn-Hansen, B., et al. (2011). Differentiation of Candida glabrata, C. nivariensis and C. bracarensis based on fragment length polymorphism of ITS1 and ITS2 and restriction fragment length polymorphism of ITS and D1/D2 regions in rDNA. *Eur J Clin Microbiol Infect Dis* 30(11)**,** 1409-1416. doi: 10.1007/s10096-011-1235-9.

Olchawa, A., Krawczyk, B., and Brillowska-Dabrowska, A. (2013). New PCR test for detection of Candida glabrata based on the molecular target chosen by the RAPD technique. *Pol J Microbiol* 62(1)**,** 81-84.

Rivero-Menendez, O., Navarro-Rodriguez, P., Bernal-Martinez, L., Martin-Cano, G., Lopez-Perez, L., Sanchez-Romero, I., et al. (2019). Clinical and Laboratory Development of Echinocandin Resistance in Candida glabrata: Molecular Characterization. *Front Microbiol* 10**,** 1585. doi: 10.3389/fmicb.2019.01585.

Simonicova, L., and Moye-Rowley, W.S. (2020). Functional information from clinically-derived drug resistant forms of the Candida glabrata Pdr1 transcription factor. *PLoS Genet* 16(8)**,** e1009005. doi: 10.1371/journal.pgen.1009005.

Taei, M., Chadeganipour, M., and Mohammadi, R. (2019). An alarming rise of non-albicans Candida species and uncommon yeasts in the clinical samples; a combination of various molecular techniques for identification of etiologic agents. *BMC Res Notes* 12(1)**,** 779. doi: 10.1186/s13104-019-4811-1.

The European Committee on Antimicrobial Susceptibility Testing (2018). *Breakpoint tables for interpretation of MICs for antifungal agents, version 9.0. EUCAST, Stokholm, Sweden 2018*

[Online]. Available: <http://www.eucast.org/astoffungi/clinicalbreakpointsforantifungals/> [Accessed June 2021 2021].

The European Committee on Antimicrobial Susceptibility Testing (2020). *Breakpoint tables for interpretation of MICs for antifungal agents, version 10.0. EUCAST, Stokholm, Sweden 2020*

[Online]. Available: <http://www.eucast.org/astoffungi/clinicalbreakpointsforantifungals/> [Accessed June 2021 2021].

Tsai, H.F., Krol, A.A., Sarti, K.E., and Bennett, J.E. (2006). Candida glabrata PDR1, a transcriptional regulator of a pleiotropic drug resistance network, mediates azole resistance in clinical isolates and petite mutants. *Antimicrob Agents Chemother* 50(4)**,** 1384-1392. doi: 10.1128/AAC.50.4.1384-1392.2006.

Xu, Y., Chen, L., and Li, C. (2008). Susceptibility of clinical isolates of Candida species to fluconazole and detection of Candida albicans ERG11 mutations. *J Antimicrob Chemother* 61(4)**,** 798-804. doi: 10.1093/jac/dkn015.

Zimbeck, A.J., Iqbal, N., Ahlquist, A.M., Farley, M.M., Harrison, L.H., Chiller, T., et al. (2010). FKS mutations and elevated echinocandin MIC values among Candida glabrata isolates from U.S. population-based surveillance. *Antimicrob Agents Chemother* 54(12)**,** 5042-5047. doi: 10.1128/AAC.00836-10.
